# Fast and sensitive detection of indels induced by precise gene targeting

**DOI:** 10.1093/nar/gkv126

**Published:** 2015-03-09

**Authors:** Zhang Yang, Catharina Steentoft, Camilla Hauge, Lars Hansen, Allan Lind Thomsen, Francesco Niola, Malene B. Vester-Christensen, Morten Frödin, Henrik Clausen, Hans H. Wandall, Eric P. Bennett

**Affiliations:** 1 Copenhagen Center for Glycomics, Departments of Cellular and Molecular Medicine and School of Dentistry, Faculty of Health Sciences, University of Copenhagen, Blegdamsvej 3, 2200 Copenhagen N, Denmark; 2 Novo Nordisk Foundation Center for Biosustainability, Danish Technical University, Lyngby, Denmark; 3 Biotech Research and Innovation Centre, University of Copenhagen, Ole Maaløes Vej 5, 2200 Copenhagen N, Denmark

## Abstract

The nuclease-based gene editing tools are rapidly transforming capabilities for altering the genome of cells and organisms with great precision and in high throughput studies. A major limitation in application of precise gene editing lies in lack of sensitive and fast methods to detect and characterize the induced DNA changes. Precise gene editing induces double-stranded DNA breaks that are repaired by error-prone non-homologous end joining leading to introduction of insertions and deletions (indels) at the target site. These indels are often small and difficult and laborious to detect by traditional methods. Here we present a method for fast, sensitive and simple indel detection that accurately defines indel sizes down to ±1 bp. The method coined IDAA for Indel Detection by Amplicon Analysis is based on tri-primer amplicon labelling and DNA capillary electrophoresis detection, and IDAA is amenable for high throughput analysis.

## INTRODUCTION

The emerging gene targeting technologies for precise editing of higher eukaryote genomes such as zinc finger nucleases (1,2) (ZFNs), transcription activator–like effector nucleases (3,4) (TALENs), RNA-guided clustered regularly interspaced short palindromic repeats (5,6) (CRISPRs) or Meganucleases (7,8), have revolutionized genome research and enabled studies previously limited to prokaryotes and yeast. These nuclease-based gene editing methods introduce double-stranded DNA breaks and lead to a variety of rearrangements at the breakpoint mediated by cellular repair events including non-homologous end-joining (NHEJ) and homologous recombination (9). In contrast to the speed by which these editing tools are being optimized and strategies for high throughput use in whole-genome screens are devised (10,11), considerably less focus are being devoted to improving capabilities for detection and characterization of the induced indels at the specific breakpoint as well as at potential off-targets. Current approaches available for identification of indels include: (i) enzyme mismatch cleavage (EMC) assays, which do not provide sensitive, reliable and accurate identification of the induced indel's (12); and (ii) Sanger or next generation DNA sequencing, which is costly, time and labour intensive and poorly suited for high throughput screening of hundreds or thousands of clones often required to select for desirable multi-allelic editing events that often occur at low frequency. Here, we report a novel strategy that combines use of a simple amplicon labelling strategy with the high throughput capability of DNA fragment analysis by automated Capillary Electrophoresis (13) for simple detection and characterization of indels induced by precise gene targeting. The strategy is coined IDAA for Indel Detection by Amplicon Analysis, and we demonstrate that IDAA is suitable for detecting indels in both cell pools with low efficiency targeting and single sorted cells. Furthermore, we show that IDAA is ideally suited for high throughput detection of indels down single base events, estimation of ‘cutting’ efficiencies of targeting tools and evaluation of off-target events at candidate loci.

## MATERIALS AND METHODS

### Precise gene targeting induced indel detection by amplicon analysis (IDAA)

IDAA primers and other primers were obtained from TAG Copenhagen A/S, Denmark (http://tagc.com/). Amplicons were fluorophore labelled by tri-primer amplification using a universal 6-FAM 5′-labelled primer FamF and primers flanking the gene editing target site of which the sense primer carried an FamF target sequence extension. All IDAA assay primers used can be seen in Supplementary Table S1. Optimal tri-primer generated amplicon yields were observed using a PCR primer ratio of 10:1:10 (FamF:XF:XR) X being either h*COSMC*, h*GALNT6*, h*KRAS*, m*Cosmc*, c*St6galnac2* or c*Cosmc (*h = human, m = murine, c = Chinese hamster ovary (CHO)). PCR was performed in 25μl, using AmpliTaq Gold (ABI/Life Technologies, USA) or TEMPase Hot Start DNA Polymerase (Amplicon, Denmark), 0,5 μM:0.05 μM:0.5 μM (FamF:F:R) primers and a touchdown thermocycling profile using an initial 72°C annealing temperature ramping down by 1 degree/cycle to 58°C, followed by an additional 25 cycles using 58°C annealing temperature. Denaturion and elongation was performed at 95°C for 45 s and 72°C for 30 s respectively. 1ul of the PCR reaction or dilutions hereof was mixed with 0.5 μl LIZ600 or LIZ500 size standard (ABI/Life Technologies, USA) and applied to fragment analysis on ABI3010 sequenator (ABI/Life Technologies, USA) using conditions recommended by the manufacturer. Raw data obtained were analysed using Peak Scanner Software V1.0 (ABI/Life Technologies, USA).

### Gene targeting plasmids and dual ZFN plasmid construction

CompoZr^®^ ZFN plasmids for h*C1GALT1C1/COSMC*, m*C1galt1c1/Cosmc* and h*GALNT6* were obtained from Sigma (Sigma-Aldrich, St. Louis, MO, USA). GeneArt^®^ TALEN c*St6galnc2* plasmids were obtained from LifeTechnologies (Thermo Fisher Scientific Inc, Waltham, MA, USA). Cas9 plasmid was codon optimized for CHO expression. Four CHO *Cosmc* gRNA targets were selected using a tool developed for CHO Cas9/gRNA target prediction (http://staff.biosustain.dtu.dk/laeb/crispy/). All Cas9 and gRNA plasmids used were generously supplied to us by Lasse Ebdrup Pedersen (Novo Nordisk Foundation Center for Biosustainability) except for pCMV-Cas9-GFP expressing gRNA for *KRAS* described previously (14). Dual *GALNT6* expression vector was constructed as follows: GFP and the two *GALNT6* ZFN1/2 sequences were fused via 2A peptide as outlined in Figure 4a. In brief, a sequence encoding GFP, Flag and nuclear localization signal (NLS) was inserted in frame into the EcoRI/KpnI site of the CompoZr^®^ *GALNT6* ZFN-2 plasmid leaving GFP fused via 2A peptide, Flag and NLS to the *GALNT6* ZFN-2 ORF as described. A full sequence of codon optimized *GALNT6* ZFN-1 (Genewiz, South Plainfield, NJ, USA) fused to sequences encoding 2A peptide, 2xmyc tag and a nuclear localization signal (NLS) was directionally inserted into the KpnI site, generating GFP-2A-*GALNT6*-ZFN-1-2A-ZFN-2 (Dual-*GALNT6*-ZFN). All plasmids were Sanger sequenced. Expression and efficient 2A peptide cleavage of ZFNs were verified by SDS-PAGE Western analysis using immune reagents to the GFP, ZFN1 (Flag-tag) and ZFN2 (myc-tag) as illustrated in Figure 4b.

**Figure 1.**
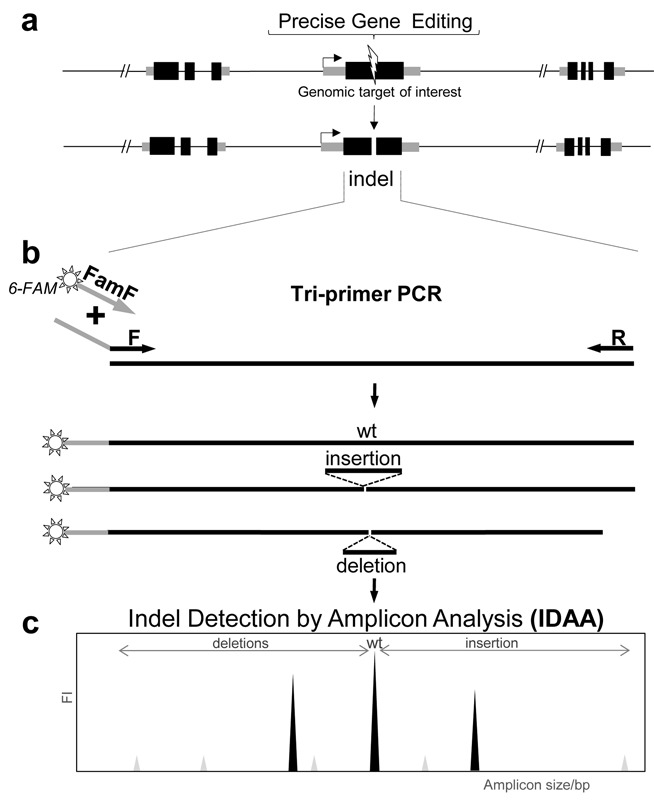
Schematic depiction of the IDAA strategy. **(a)** Precise gene targeting creates double-stranded breaks that through NHEJ introduce indels at the target site. **(b)** Tri-primer PCR of the target region accomplished by use of target specific primers (F/R) flanking the target site and a universal 5′-FAM labelled primer (FamF) specific for a 5′-overhang sequence attached to primer F. Tri-primer PCR results in FAM amplicon labelling. **(c)** Fluorescently labelled amplicons containing the indels are detected by fragment analysis. Axis represent fluorescence intensity (FI) and amplicon size in base pairs.

**Figure 2.**
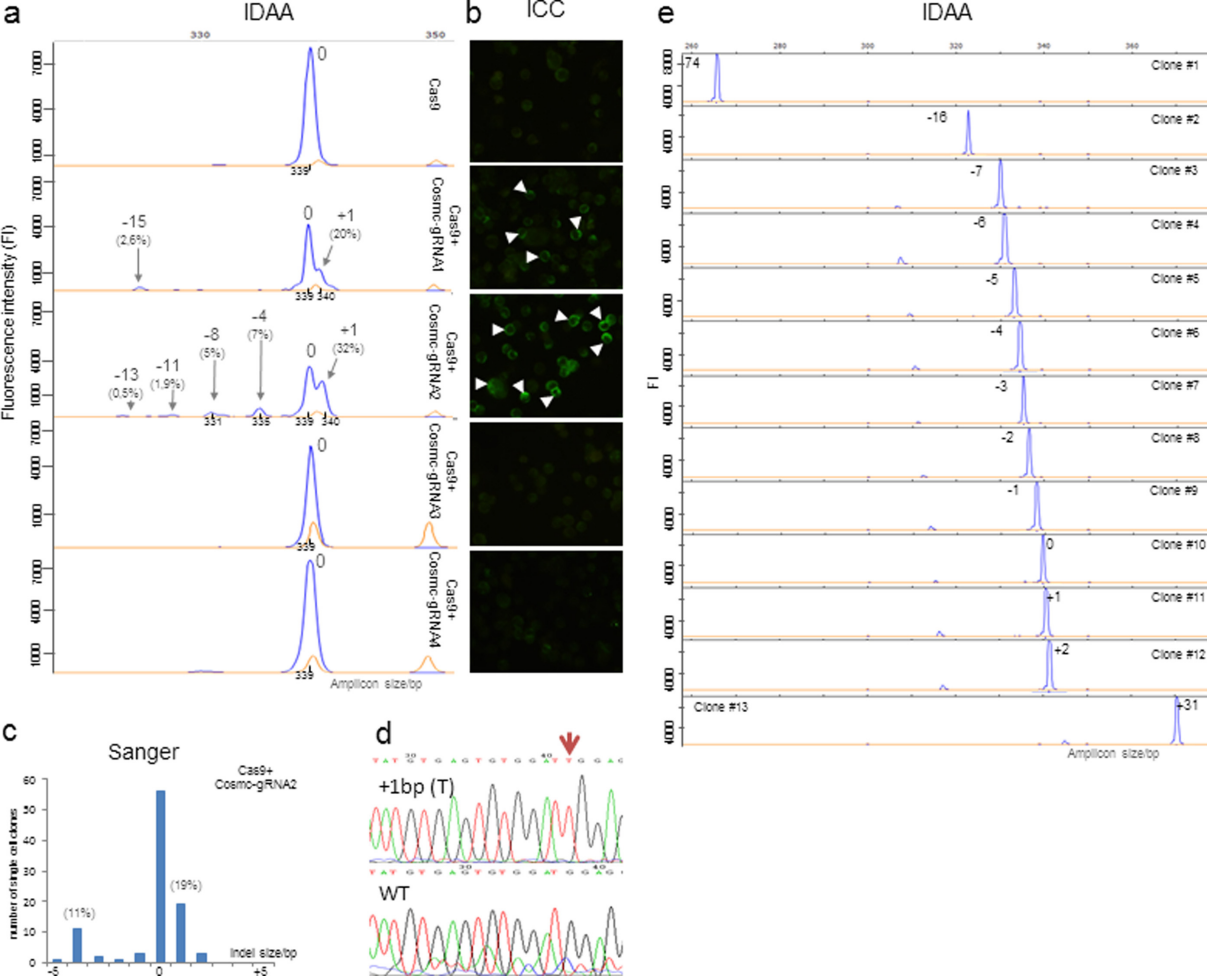
Evaluation of IDAA for detection of indels in gene targeted CHO cell pools and derived single clones. **(a)** IDAA of a pool of CHO cells at day two after nucleofection with Cas9 and four different gRNA designs targeting *Cosmc*. Shown from top are IDAA of cell pools transfected with Cas9 alone, with gRNA1, gRNA2, gRNA3 and gRNA4 (bottom). The position of the unmodified wild-type amplicon peak is indicated (0) and amplicon sizes (in bp) as determined by Peak Scanner software are indicated below peaks. Indel sizes determined (in bp) are shown above the most prominent peaks together with cutting efficiencies (calculated from peak area relative to total peak area) in percentage. Total cutting efficiency for gRNA1 and gRNA2 were estimated to 23% and 46%, respectively, while gRNA3 and gRNA4 were inactive. The relative frequency of indels produced by gRNA2 was confirmed by MiSeq deep sequencing (Supplementary Figure S6a) which further revealed that the predominant +1 insertion was an thymine insertion at a position three bases upstream of the PAM sequence. The GSLIZ500 standard peaks are shown in orange. **(b)** Comparative ICC analysis of the corresponding cell pools shown in (a) seven days after nucleofection with a monoclonal antibody (5F4) detecting the de novo induction of truncated O-glycans as a result of complete inactivation of *Cosmc* (arrow heads indicate single positive cells in pool). **(c)** Analysis of indels introduced by Sanger sequencing showing distribution in the −5 bp to +5 bp range of individual single cell clones. **(d)** Single cell clone Sanger confirmation of the predominant +1 bp indel identified by IDAA. **(e)** Representative IDAA analysis of single cell clones showing the 1 bp resolution power of IDAA.

**Figure 3.**
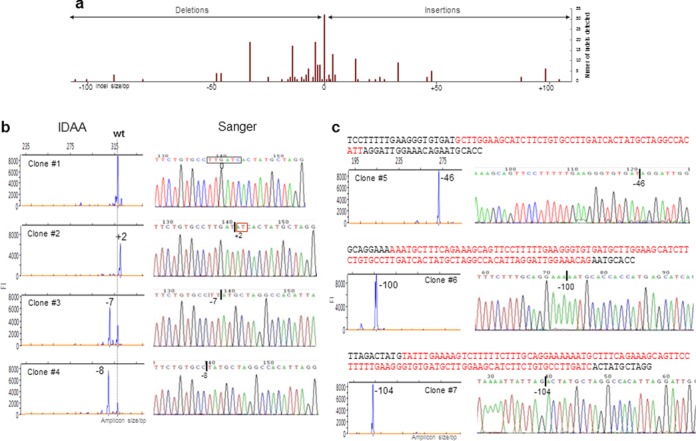
Distribution of indels found in *Cosmc*-ZFN targeted single cell FACS sorted MC57 clones. **(a)** The wild-type allele amplicon is denoted 0 (0 bp indel), and 81 clones with indels of varying sizes were identified. **(b)** Comparative IDAA and Sanger sequencing of representative clones. ZFN cutting site is shown boxed in wt Sanger panel. Indels determined by IDAA are indicated in bp sizes at peaks. For Sanger sequencing the position of the deletion is indicated with a line in the sequence, insertion is boxed in red. **(c)** For the larger deletions detected,the sequence of the targeted region is shown above the IDAA and Sanger panels, with the deleted sequence shown in red. For Sanger sequencing the position of the deletion is indicated with a line in the sequence.

**Figure 4.**
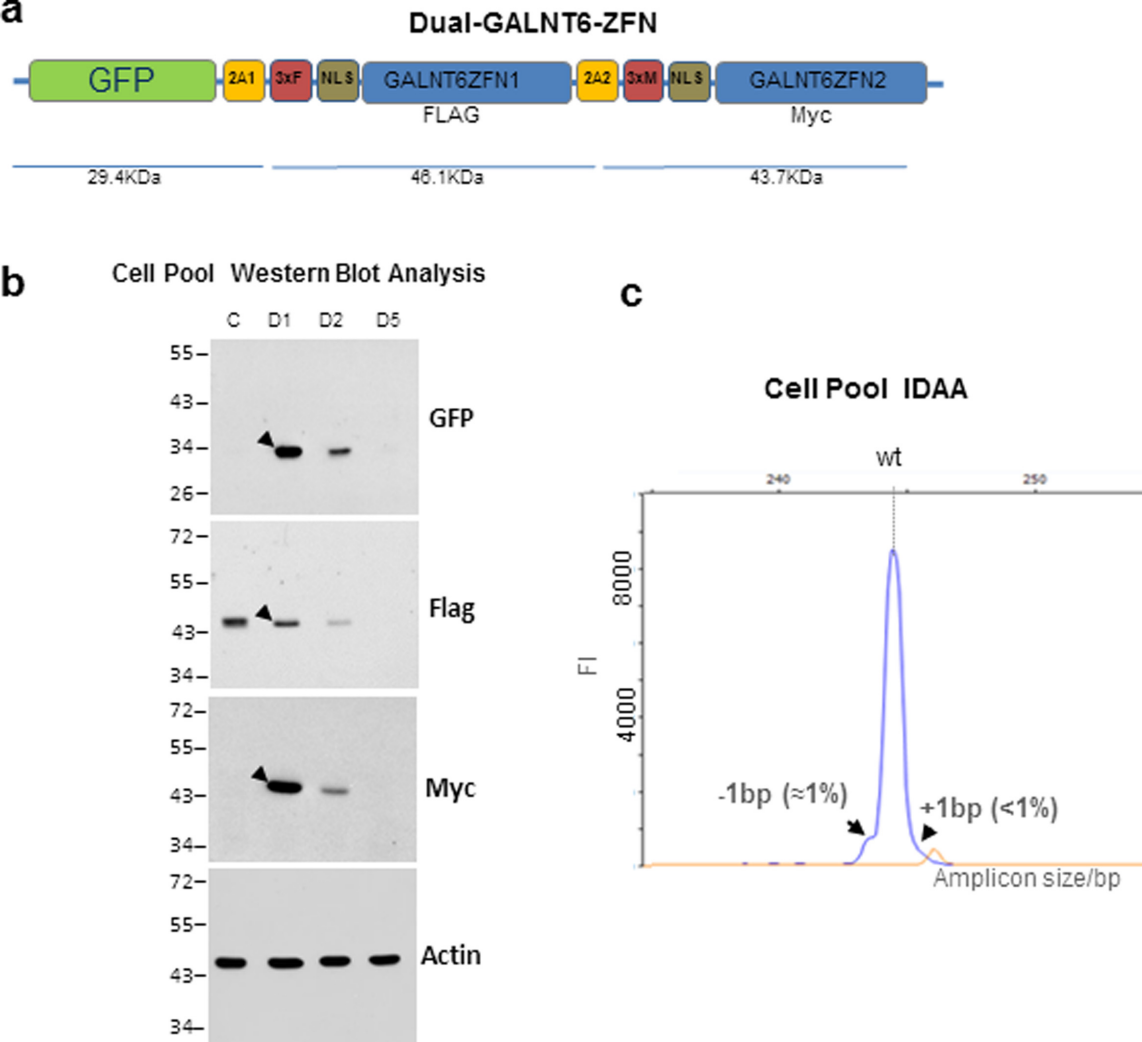
Schematic depiction of the labelled ZFN targeting vector for GALNT6 (Dual-*GALNT6*-ZFN). **(a)** GFP, 2A peptides (2A1 and 2A2), 3xFLAG (3xF), nuclear localization signal (NLS), ZFN1 and ZFN2 for *GALNT6* (GALNT6ZFN1 & 2), and 3xMyc (3xM) are indicated. **(b)** SDS-PAGE Western blot analysis of K562 cells harvested day 1 (D1), 2 (D2) and 5 (D5) after nucleofection with the Dual-*GALNT6*-ZFN plasmid targeting vector. Blots were reacted with anti-GFP, anti-FLAG, anti-Myc or anti-actin antibodies as indicated. Arrow heads indicate reactive bands with expected mobilities. For control transfections (C) K562 cells were transfected with monomeric Flag-tagged original ZFNs (Sigma-Aldrich) and harvested day 1. **(c)** IDAA of a HepG2 cell pool at day 2 post nucleofection with Dual-*GALNT6*-ZFN. The low efficiency and prevalence of ±1 bp indels were confirmed by MiSeq deep sequencing (Supplementary Figure S6b). Indel percentages (shown in parenthesis) were calculated from peak areas of the summed indel peaks relative to the total peak area.

### Cell culture, transfections and FACS sorting

HeLa, DE4, HEK293AC2, mouse MC57 cells were cultured in Dulbecco's modified Eagle's medium with 10% FBS and 1% L-glutamine, and K562 cells were cultured in Iscove's modified Dulbecco's medium, 10% FBS and 1% L-glutamine. CHO cells were cultured in ex-Cell-CD media (Sigma Aldrich, USA) with 2% L-glutamine. Cells were nucleofected using solution kits T and V (K562) (Lonza, USA) and a Amaxa^®^ Cell Line Nucleofector^®^ device as previously described (15,16) using protocols provided by Lonza (http://www.lonzabio.com/resources/product-instructions/protocols/). In brief, 1 × 10^6^ cells were transfected with 2 μg of ZFN or TALEN plasmid pairs or 2 μg of Dual-*GALNT6*-ZFN. For CRISPR/Cas9 CHO *Cosmc* targeting, 2 μg Cas9 and gRNA plasmids were nucleofected, and for pCMV-Cas9-GFP expressing *KRAS* gRNA 2 μg plasmid was used. Cells were exposed to a cold shock 30°C for 2 days post-transfection, and incubated one day at 37°C after which DNA of the cell pool was prepared using Nucleospin kit as recommended by the supplier (Machery-Nagel, USA).

For consecutive targeting of the *KRAS* locus, K562 cells were subjected to fluorescence-activated cell sorting (FACS) 3 days after nucleofection for isolation of the 2% most highly GFP fluorescent cells that were then cultured for about 1 week. Thereafter, an aliquot of the cell pool was analysed by IDAA (first hit), whereas the rest of the cells were subjected to another two rounds of nucleofection and FACS to produce second and third hit pools, respectively, Furthermore, after the third hit, cells were also single-cell plated in 96-well plates and expanded to clonal cell lines.

### Direct sanger sequencing

Expanded single cell clones were lysed in the wells using QuickExtract DNA extraction solution (Sigma-Aldrich, USA), 1 μl lysate was used for target region amplification. Amplified products were band purified using Qia-mini elute purification (Qiagen Inc, USA) and used for Topo-ligation into pCR4-Topo vector (Invitrogen/Life Technologies, USA), transformed into MegaXcells (Invitrogen/Life Technologies, USA) and LB-Streptomycin/Carabenicillin plated. A custom based direct sequencing protocol was developed by which large sized single cell colonies were boiled in 10 μl TE for 10 min and 5 μl hereof added to BigDye.3.1 reaction (ABI/Life Technologies, USA) and sequenced using 45x sequencing cycles (BGI Europe, Denmark).

### T7EI-nuclease assay

Endonucleolytic heteroduplex DNA cleavage analysis was performed using T7-nuclease-I (New England Biolabs, USA) as recommended by the supplier. In brief, heteroduplex and/or perfect match amplicons were incubated with 1 μl T7nuclease in a 20 μl volume at 37°C for 1 h, followed by 3% agarose gel GelStar (Lonza, USA) analyses.

### SDS-PAGE Western blotting and immunocytochemistry

K562 cells were nucleofected as described above. After 1, 2 or 5 days, aliquots of the cells were harvested and lysed in SDS-PAGE sample buffer. The cell lysates were normalized for protein content and equal amounts of protein were subjected to immunoblotting. Blots were incubated with primary antibodies to the proteins indicated (beta-Actin: Abcam, cat. number: ab8226; c-Myc: Santa Cruz Biotechnology, cat. number: sc-40; Flag: Sigma-Aldrich, cat. number: F3165) followed by HRP-conjugated secondary antibodies (Dako, Denmark), both for 1 h at room temperature and finally developed with ECL (Pierce/Thermo Scientific, USA). For immunocytochemistry (ICC), CHO cells were fixed and stained on Teflon coated slides as previously described (17). In brief, cells were dried on slides and incubated overnight, 4°C, with the monoclonal antibody 5F4, followed by secondary anti-mouse-Ig-FITC incubation (Dako, Denmark), visualization and imaging by fluorescence microscopy as previously described (17).

## RESULTS AND DISCUSSION

### IDAA for estimating cutting efficiencies for CRISPR/Cas9-gRNA designs

The amplicon labelling strategy for evaluation of PCR product sizes was originally introduced by Oetting *et al*. (18), using polyacrylamide gel electrophoresis to resolve larger size differences in products. The strategy was further improved by Schuelke (19) using capillary electrophoresis for resolving smaller size differences down to ±2 bp of microsatellite repeats and a similar strategy was also used by Mellersh *et al*. (20). Here, we built further on this strategy and advanced the detection resolution down to a single base pair size discrimination. We used a single-step tri-primer PCR setup with a universal 6-FAM 5′-labelled primer (FamF) designed to a specific extension of the forward target specific primer, which enables one-step fluorophore labelling of amplicons derived from any given target using a universal amplification condition (Figure 1a). In contrast to previous M13 based primer designs (18,19), we used a non-M13 based primer design (5′-AGCTGACCGGCAGCAAAATTG-3′) increased in sequence length (21 versus 18 bases) and G/C content (52% versus 50%) for 6-FAM labelling, which enables use of increased assay stringency conditions and results in high product quality with single peak generation. The tri-primer amplification assay was standardized and optimized for general use in detecting a broad range of targets, and optimal amplicon yields were obtained using 10:1:10 molar ratios of 5′-labelled forward primer:unlabelledforward primer:reverse primer (Figure 1b and Supplementary Table S1). More than 50 different gene targets in different species have been profiled using these assay conditions. The fluorophore labelled amplicons can easily be detected with great sensitivity and with accurate size determination down to ±1 bp using standard DNA fragment analysis by capillary electrophoresis methodology (13) (Figure 1c). Importantly, the method applied enabled unbiased amplicon size discrimination with crude cell lysates as template source (Figure 3 and Supplementary Figures S1 and S2). To demonstrate the applicability of the IDAA strategy in nuclease-based genome editing, we first demonstrate its use for evaluating cutting efficiencies of CRISPR/Cas9 targeting using four different gRNA designs (Figure 2a). We targeted the *Cosmc* gene (X-linked) in CHO cells, because CHO only has one *Cosmc* allele and we have a very reliable phenotypic screen for knockout of *Cosmc* function (15). We found consistent targeting efficiencies of total cell pools when using either IDAA or phenotypic screening 3d after transfection with the constructs (Figure 2b). The IDAA analysis of the total cell pools and Sanger sequenced single cell clones furthermore revealed that indels ranged from +1 to -13 with an apparent frequency of <1% to a few percent for most of them (Figure 2a and c). Notably the identity of the predominant +1 (approximate frequency 20% and 32% for gRNA1 and gRNA2 respectively) insertion was validated by Sanger and found to be a T/A base-pair insertion (Figure 2d).

### Comparison of IDAA against Sanger sequencing and enzyme mismatch cleavage assay

We next demonstrated that IDAA is amenable for high throughput screening of targeted individual cell clones with a discrimination power down to a single base indel. We used the same CRISPR/Cas9 *Cosmc* gRNA2 targeted pool for single cell cloning shown in Figure 2a, and analysed approximately 200 independent single cell clones by Sanger and IDAA. Notably, the −5 bp to +5 bp indel distribution obtained by MiSeq next generation deep sequencing (Supplementary Figure S6a) and Sanger was similar to the IDAA distribution on the cell pool (Figure 2a and c). A variety of distinct clones with indels ranging from −74 bp to +31 bp were readily identified by IDAA, as exemplified in (Figure 2e), which also illustrates the 1 bp resolution power of IDAA for indel genotyping of clones and the ease by which clones harbouring reading frame-disrupting indels can be identified. The indels were confirmed by Sanger sequencing as shown for the predominant +1 insertion identified (Figure 2d).

To corroborate the usability of IDAA for multi-allele gene targeting, bi-allelic CHO *ST6galnac2* and tri-allelic human K562 *KRAS* were targeted with TALEN and CRISPR/Cas9 nucleases respectively. The IDAA results for TALEN CHO *St6galnac2* two days after transfection clearly detected indels in the cell pool and successful bi-allelic targeting was obtained in a substantial fraction (48%) of FACS single cell clones analysed (Supplementary Figure S1a and b). For CRISPR/Cas9 human K562 *KRAS* targeting, FACS for the 2% most highly fluorescent cells generated a cell pool (first hit) in which IDAA revealed indels in the large majority of alleles and only a minor wt peak (Supplementary Figure S2a). When this pool was subjected to further two consecutive rounds of targeting and FACS, IDAA revealed near-complete modification of the *KRAS* locus, since the wt peak was hardly detectable. By contrast, these large modification rates were greatly underestimated by EMC assay (Supplementary Figure S2b). Sanger sequencing and IDAA analysis of 96 single cell clones isolated from the third hit pool confirmed the indel profile obtained by IDAA on the third hit cell pool and successful tri-allelic targeting in 99% of the clones was detected by IDAA (only 1 wt allele was detected) (Supplementary Figure S2c and d). The IDAA clone analysis in Supplementary Figure S1b and S2d illustrate the easy by which clones harbouring reading frame-disrupting indels in all alleles present can be identified. By contrast, no such information can be derived from EMC assay, which even fails to detect complete allele modification in clones, as illustrated in Supplementary Figure S2b.

We further showed that the IDAA strategy is ideal for ZFNs, which generally have lower cutting efficiencies. We first tested a ZFN with medium cutting efficiency (18% evaluated by an EMC assay, Sigma-Aldrich) targeting the *Cosmc* gene in a murine cell line (MC57) (Figure 3). We used a GFP-tagged ZFN approach that enables FACS sorting for enrichment of targeting events as recently described (14). A total of 81 single cell clones derived from 192 FACS seeded wells were tested by IDAA, and we found complete correlation between the IDAA results and Sanger sequencing. We then tested a ZFN with low cutting efficiency (2.8% as determined by an EMC assay, Sigma-Aldrich) targeting the human *GALNT6* (21) gene in human HepG2 cells (Figure 4). For this we used a novel dual expression plasmid encoding both ZFNs fused to GFP (Dual-*GALNT6*-ZFN). We used IDAA to analyse the cell pool at day two post-transfection, and we could demonstrate that the predominant targeting events were ±1 bp indels detectable with around only 1% frequencies. These predominant low targeting events could also be detected by MiSeq deep sequencing (Supplementary Figure S6b), but such patterns of targeting events with predominantly small indels are not expected to be detectable by the traditional EMC assays. To test this hypothesis, we performed a head-to-head comparison of IDAA with the most commonly used indel detection assay. The EMC assay has been shown to display a preference for heteroduplex DNA formed by larger deletions rather than single 1 base indels (12), and it does not provide information on the nature of the indels and types of alleles present. We first demonstrated that the EMC assay using T7 endonuclease I easily detected a large deletion, in this instance induced by a ZFN (Supplementary Figure S3a). We next tested the EMC assay on a *COSMC* targeted cell clone with only one allele and possessing a single base indel. In this case amplicons derived from the HeLa targeted *COSMC* allele (22) and wild-type HeLa cells were mixed and analysed, and we confirm that whereas larger DNA deletions are easily detected by EMC, a single base indel is not (Supplementary Figure S3b).

### IDAA for detection of candidate off-target indels

Finally, we demonstrate that IDAA is ideally suited for fast and simple screening for indels in candidate off-target genes identified by target sequence similarity, which is a major concern for all precise gene editing strategies. We first screened the CHO genome for the most likely off-target sites for gRNA2 targeting *Cosmc*, and identified a total of 189 potential off-targets with two to four bp mismatches (Supplementary Figure S4a). Detailed mismatch distribution for the top 21 off targets are shown in Supplementary Table S2. We tested the most likely off-target (2 mismatches, no candidate genes for gRNA2 with only one mismatch were found) in 10 independent *Cosmc* Cas9/gRNA2 targeted single cell clones by IDAA and we could demonstrate complete absence of off-target events in all clones (Supplementary Figure S4b). We next screened additional 20 most likely off-targets (3–4 mismatches) in all 10 clones, and again confirmed complete absence of off-target events in all 10 clones (Supplementary Figure S5). It should be stressed that our analysis was conducted on independent cell clones as opposed to recent reports where rare off-target effects were observed in cell pools (10,11).

## CONCLUSION

In conclusion, the IDAA strategy presented here enables sensitive, precise and reliable identification of indels in a high throughput mode providing detailed information of cutting efficiency, size and nature of allelic variants generated by any of the precise gene editing technologies. The IDAA strategy is user friendly and easily implemented in any standard laboratory, and we expect that this screening tool will greatly advance implementation and use of precise gene targeting.

## SUPPLEMENTARY DATA

Supplementary Data are available at NAR online.

SUPPLEMENTARY DATA
