# Supplementary material for: Fast and sensitive detection of indels induced by precise gene targeting
Source: Nucleic Acids Res. 2015 Mar 9;43(9):e59. doi: 10.1093/nar/gkv126 (PMC4482057; doi:10.1093/nar/gkv126)
Supplement: SUPPLEMENTARY DATA [file supp_43_9_e59__index.html]

Fast and sensitive detection of indels induced by precise gene targeting — SUPPLEMENTARY DATA 

# Fast and sensitive detection of indels induced by precise gene targeting

## SUPPLEMENTARY DATA

**Files in this Data Supplement:**

- SUPPLEMENTARY DATA
