# Supplementary material for: Fast and sensitive detection of indels induced by precise gene targeting
Source: Nucleic Acids Res. 2015 Mar 9;43(9):e59. doi: 10.1093/nar/gkv126 (PMC4482057; doi:10.1093/nar/gkv126)
Supplement: SUPPLEMENTARY DATA [file supp_gkv126_revised_Supplementary_Figures_NAR_final.pdf]

Supplementary information for

Fast and Sensitive Detection of Indels Induced by Precise Gene Targeting

Zhang Yang [1,2], Catharina Steentoft[1], Camilla Hauge[1,2,3], Lars Hansen[1], Allan Lind-Thomsen[1], Francesco Niola[3], Malene B. Vester-Christensen[1], Morten Frodin[3], Henrik Clausen[1,2], Hans H. Wandall[1] and Eric P. Bennett[1]*

[1] Copenhagen Center for Glycomics, Departments of Cellular and Molecular Medicine and School of Dentistry, Faculty of Health Sciences, University of Copenhagen, Blegdamsvej 3, 2200 Copenhagen N, Denmark

[2] Novo Nordisk Foundation Center for Biosustainability, Danish Technical University, Lyngby, Denmark

[3] Biotech Research and Innovation Centre, University of Copenhagen, Ole Maaløes Vej 3, 2200 Copenhagen N, Denmark

*To whom correspondence should be addressed.

This file includes:

Supplementary Tables 1-2

Supplementary Figures 1-6

Supplementary Tabel I

Gene	primer	sequence*
FAMF		5’-AGCTGACCGGCAGCAAAATTG-3’
hCOSMC	COSMCFAMFOR	5’-AGCTGACCGGCAGCAAAATTGAGGGAGGGATGATTTGGAAG-3’
	COSMCF	5’-AGGGAGGGATGATTTGGAAG-3’
	COSMCR	5’-TTGTCAGAACCATTTGGAGGT-3’
mCosmc	COSMCFAMFOR	5’-AGCTGACCGGCAGCAAAATTGGCATGTGGACCTTTGGTTTT-3’
	COSMCF	5’-GCATGTGGACCTTTGGTTTT-3’
	COSMCR	5’- TTACTGAGCTCCATGCGTTC-3’
cCosmc	COSMCFAMFOR	5’-AGCTGACCGGCAGCAAAATTGGGATCCATCGCAGCCTTTCT-3’
	COSMCF	5’-GGATCCATCGCAGCCTTTCT-3’
	COSMCR	5’-ACTACCTGGTTCGGGTGGTT-3’
	gRNA1	5’-GAAAAGTGTCCTGAACAAGG-3’
	gRNA2	5’-GAATATGTGAGTGTGGATGG-3’
	gRNA3	5’-GCAGTCTGCCTGAAATATGC-3’
	gRNA4	5’-GAAATATGCTGGAGTATTTG-3’
	MISEQCHOCOSMCZFNF	5’-GCTGACCGGCAGCAAAATTGAGTGTGGATGGAGGGATTGTC-3’
	MISEQCHOCOSMCZFNR	5’-TCCAGCATATTTCAGGCAGAC-3’
	MISEQCHOCAS9F	5’- AGCTGACCGGCAGCAAAATTGCGCCCCAGTACTTTTGCTGTGATTG-3’
	MISEQCHOCAS9R	5’-ATTCATCCCACCTTGTTCAGG-3’
hGALNT6	T6FAMFOR	5’-AGCTGACCGGCAGCAAAATTGGGAGGCCATGAACAACCTTA-3’
	GALNT6F	5’-GGAGGCCATGAACAACCTTA-3’
	GALNT6R	5’-GTCTCCCCACAAGGACTCTG-3’
	MISEQT6F	5’-AGCTGACCGGCAGCAAAATTGTCCAAATCAGGGCTCCAGAA-3’
	MISEQT6R	5’-GGCAAAGGCATTGAAACAGTG-3’
cST6GALNACII		
	ST6IIFOR	5’-AGCTGACCGGCAGCAAAATTGAGGCAGAAGACAGGGGAGAA-3’
	ST6IIR	5’- ATAGGCTCCCAGGAGACACA-3’
hKRAS	KRASFOR	5’-AGCTGACCGGCAGCAAAATTGAAAAGGTACTGGTGGAGTATTTGA-3’
	KRASR	5’-TCATGAAAATGGTCAGAGAAACC-3’
“OFF TARGET” PRIMERS^:		
Forward	OT1	5’-AGCTGACCGGCAGCAAAATTGCCCGGTGCCTTCTTAGATTT-3’
	OT2	5’-AGCTGACCGGCAGCAAAATTGAGAGGCAGGTGGATCTCTGA-3’
	OT3	5’-AGCTGACCGGCAGCAAAATTGGGCCTGATGCTCTGATGATT -3’
	OT4	5’-AGCTGACCGGCAGCAAAATTGAATATGGAGGGCTCAAGGTG -3’
	OT5	5’-AGCTGACCGGCAGCAAAATTGGGTGGTGACAGGTTGAACATC -3’
	OT6	5’-AGCTGACCGGCAGCAAAATTGAGCCCCTCCACGTCATTT -3’
	OT7	5’-AGCTGACCGGCAGCAAAATTGGACCCTGTCTCCAAAGCAAG -3’
	OT8	5’-AGCTGACCGGCAGCAAAATTGAGGGGCCATCTGTTAGATCC -3’
	OT9	5’-AGCTGACCGGCAGCAAAATTGGATTATGCATATGATGTTTGTGC -3’
	OT10	5’-AGCTGACCGGCAGCAAAATTGTGTTGATCTAAACCAGTGGAACA -3’
	OT11	5’-AGCTGACCGGCAGCAAAATTGTGAGATGCAGAGTGGTGGAG -3’
	OT12	5’-AGCTGACCGGCAGCAAAATTGTCCCATCCGATAAGGACTTG -3’
	OT13	5’-AGCTGACCGGCAGCAAAATTGTGAATGACCCCTGTCTGAAA -3’
	OT14	5’-AGCTGACCGGCAGCAAAATTGTGTGTGTGAAGGTTTAAGTGCAT -3’
	OT15	5’-AGCTGACCGGCAGCAAAATTGTTTTGGCTGCACAATCTCAG -3’
	OT16	5’-AGCTGACCGGCAGCAAAATTGGCCTGCCATGTGTTTCTTTC -3’
	OT17	5’-AGCTGACCGGCAGCAAAATTGTTTTGTGTGCCTGTCTGTGT -3’
	OT18	5’-AGCTGACCGGCAGCAAAATTGCACCAGGTCTCAAACACCAA -3’
	OT19	5’-AGCTGACCGGCAGCAAAATTGCATTTCCAGTCACTGACACCA -3’
	OT20	5’-AGCTGACCGGCAGCAAAATTGTCCCATGGTTGGTTAGCTTG -3’
	OT21	5’-AGCTGACCGGCAGCAAAATTGCAGAGGGCCACCAATCTTTA -3’
Reverse	OT1	5’-AAGTGCCTTGACTCTCTCCGTA -3’
	OT2	5’-CTGGTTCTTGGGTCCTCTTG -3’
	OT3	5’-CCCTGAGGCTTTCTGTGTTT -3’
	OT4	5’-CCCAGTACTCTCTCCCTCCA -3’
	OT5	5’-AGGGCAGGGGACTGAAATAA -3’
	OT6	5’-TGTGCCCTCACAGTAACACC-3’
	OT7	5’-GGCACAAACACCAACACTCA-3’
	OT8	5’-TGCCTTCCTCTCCTTTGTTG-3’
	OT9	5’-TGAACCTGTCTCAAGGTGGA-3’
	OT10	5’-TCCTCTTGGGGCTTAAACAA-3’
	OT11	5’-TATGAGCAGTCTCCCCAGGT-3’
	OT12	5’-CTTGACAAAGCAGCCAACTG-3’
	OT13	5’-GCTTAGAGCTGCTTCCCAGA-3’
	OT14	5’-TTCCAAAACAGAAGGGCATC-3’
	OT15	5’-TCCTTTATTCCCTCTAACCCATC-3’
	OT16	5’-ACCCCTGGAGTCATGAGACA-3’
	OT17	5’-CTGGGTCGAGGAAAGAAGAT-3’
	OT28	5’-TGGTTTGTGCAAGATTCCAA-3’
	OT19	5’-GCATCGGTGATATTAATTCTTCCT-3’
	OT20	5’-AATCATATAGAGGCCATGACTTTGA-3’
	OT21	5’- GGCCAACCTCCTATTGACAG-3’

*FAMFOR extension is underlined and represents a modified design previously described(20). Species specificity of the primers is indicated with; h for human, c for CHO and m for mouse

^”Off target” primers and amplification primers listed have been designed using Primer3 software(23).

Supplementary Table II "Off target" mismatch distribution at top 24 sites

		*		strand	gRNA gene target´s
wt	:	GAATATGTGAGTGTGGATGGAGG	: 23	-	Cosmc
1	:	GAATATGTGAGTGTGCAGGGAGG	: 23	-	ras-related protein Rab-33A-like
2	:	GAATATGTGTGTGGGGAGGGAGG	: 23	-	intraflagellar transport protein 81
3	:	GACTCTGTGTGTGTGGATGGAGG	: 23	-	unplaced genomic scaffold 1437
4	:	GTATATGTGAGTGTGTGTGGAGG	: 23	-	unplaced genomic scaffold 1699
5	:	GAATATTTGTGTGTGGATTGAGG	: 23	+	unplaced genomic scaffold 300
6	:	GAAGCTCTGAGTGTGGATGGAGG	: 23	-	unplaced genomic scaffold 1586
7	:	GTATATGTGAGTGTGCATGCAGG	: 23	+	unplaced genomic scaffold 867
8	:	GAATTTGTGAGTGTGGGTGTAGG	: 23	-	unplaced genomic scaffold 3676
9	:	GAAAAGGTGAGTGTGGATTGAGG	: 23	+	unplaced genomic scaffold 1413
10	:	GACTGTGTGAGTGTGAATGGAGG	: 23	+	unplaced genomic scaffold 6488
11	:	GAAGCTCTGAGTGTGGATGGAGG	: 23	-	unplaced genomic scaffold 3651
12	:	GAAAATGTGTGTGTGTATGGGGG	: 23	-	hypothetical protein LOC100752970
13	:	GAGGAAGTGAGTGTGGGTGGAGG	: 23	+	prospero homeobox protein 1
14	:	GAATCTGGGAATGTGAATGGAGG	: 23	+	cadherin-13
15	:	GTATATGTGGGTGTGTGTGGAGG	: 23	-	polycystic kidney disease protein 1-L2
16	:	GTATGTGTGTGTGTGGATGGTGG	: 23	+	S-adenosylmethionine mitochondrial protein
17	:	GAATTTGAGAGTGGGGATGTAGG	: 23	+	bone morphogenetic protein 2-like
18	:	GGATATGAGAGTATGGAGGGAGG	: 23	+	interferon alpha/beta receptor 2-like
19	:	GTAAAGGTGAGTGTGCATGGAGG	: 23	+	disabled homolog 1-like
20	:	GAATGTGTGTGTGTGGAGGGGGG	: 23	+	disabled homolog 1-like
21	:	GTGTGTGTGTGTGTGGATGGAGG	: 23	-	E3 ubiquitin-protein ligase RNF216-like
22	:	GAATGTGTGTGTGTGCATGTAGG	: 23	+	mitogen-activated protein kinase MLT-like
23	:	GGATATGAGGGTTTGGATGGAGG	: 23	-	importin-11
24	:	GAATATATGTGTGTTGGTGGAGG	: 23	-	S1 RNA-binding domain-containing protein

*indicates the last base in gRNA preceeding the PAM seed sequence

Supplementary Figure 1 │ IDAA of TALEN bi-allelic CHO *St6Galnac2* targeting.

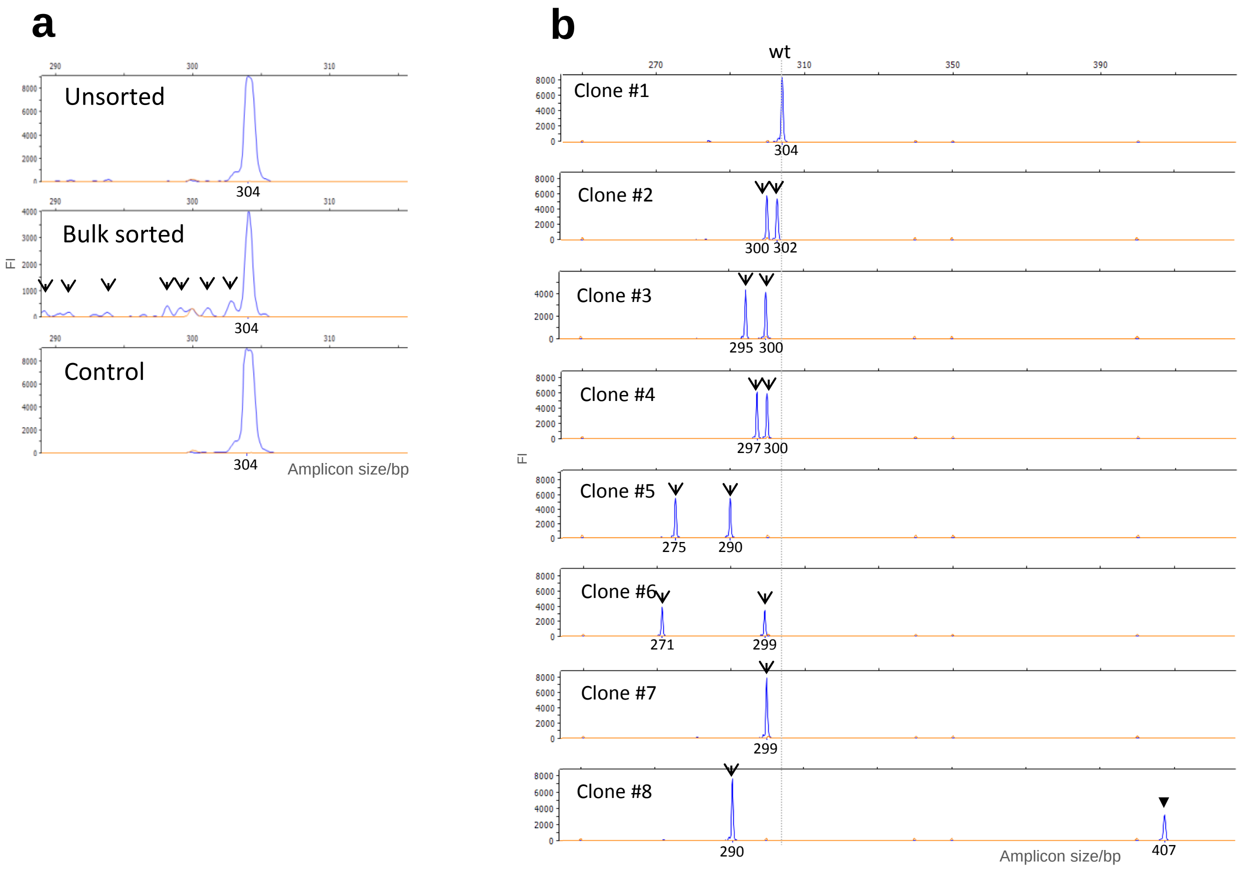

Bi-allelic CHO *St6galnac2* TALEN targeting evaluated by IDAA: Bi-allelic CHO *St6galnac2* was targeted using a GFP tagged custom designed TALEN (see material and methods) directed to the target sequence; TCCAGAGGTCTTGAGTGAAgaggctgccccatagCAGCACTGGGGTGGAGAGA (TALEN binding site sites shown in upper case, linker in lower case). **Panel a:** IDAA result of day 2 post transfection unsorted cells, FACS bulk sorted cells or control cells. Note the increase in indels detected for the bulksorted cells (arrow heads) compared to the unsorted cells**. Panel b:** IDAA results for 8 FACS sorted independent single cell clones displaying a variety of different indels. Upper panel represents wt allele, deletions are shown by open arrow heads and insertions by filled arrow heads. A total of 54 single sorted clones were analyzed, 26% (14/54) wt, 26% (14/54) mono allelic targeted and 48% (26/54) bi-allelic targeted.

Supplementary Figure 2 | IDAA of CRISPR/Cas9 tri-allelic human K562 *KRAS* targeting.

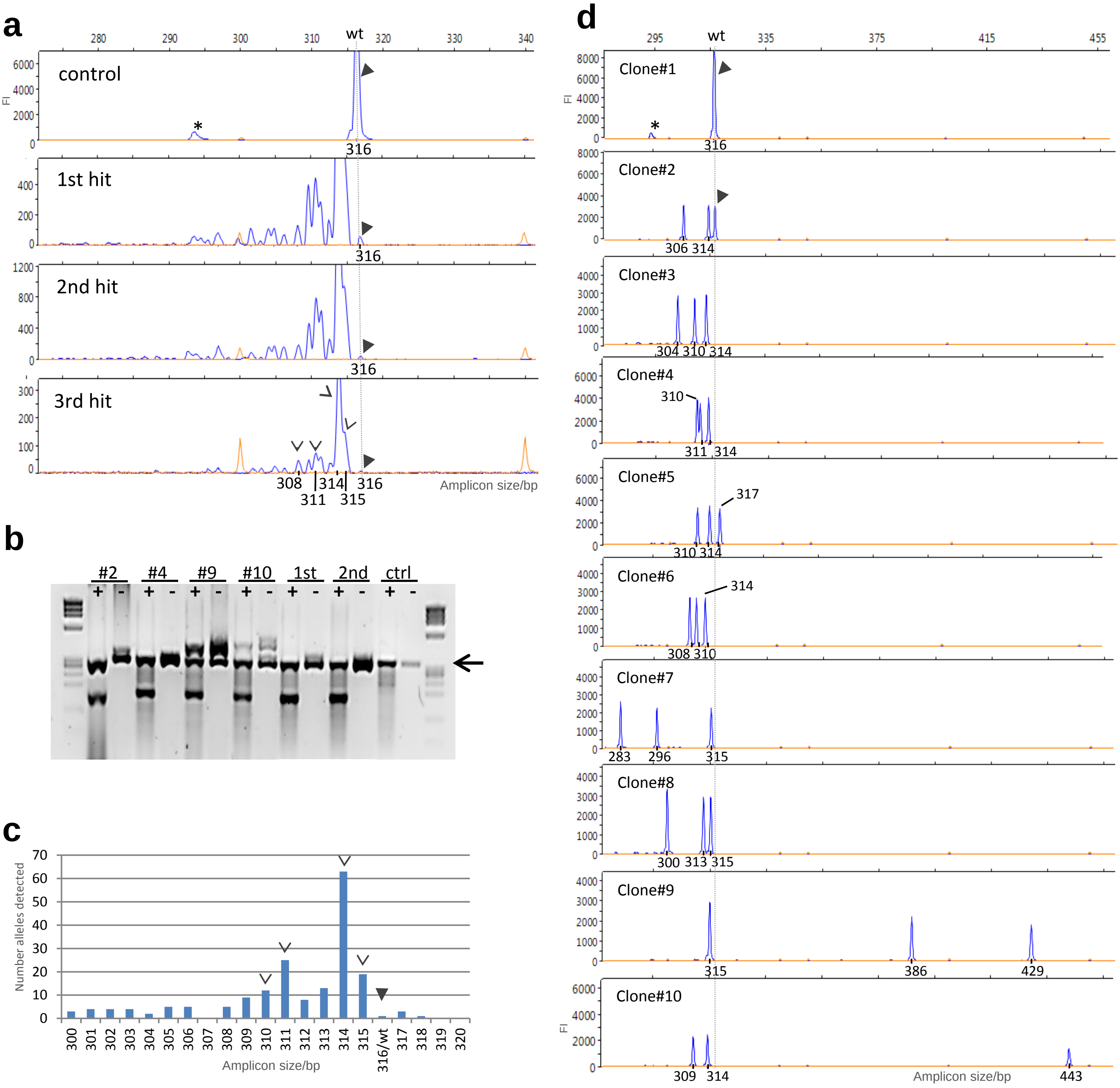

Tri-allelic K562 *KRAS* CRISPR/Cas9 targeting evaluated by IDAA: Tri-allelic K562 *KRAS* gene was targeted using a plamsid expressing Cas9-2A-GFP and *KRAS* gRNA as previously described(14) and indels were detected after 3 consecutive rounds *KRAS* CRISPR/Cas9 targeting followed by FACS sorting of GFP positive cells, cell expansion and re-transfection (1st, 2nd and 3rd hit of cells). **Panel a:** IDAA result of 1st, 2nd and 3rd rounds of transfected cells day 2 post transfection. Untransfected cell control is shown in upper panel. The appearance of an unspecific minor peak observed in this assay is indicated by an asterix. The position of the wt peak is indicated by filled arrow heads. Notably the wt peak is significantly diminished after the 3rd hit. Dominant peaks are marked by open arrow heads. Amplicon peaks are shown in blue and the LIZ500 standard in orange. **Panel b:** EMC/T7-assay results of representative 3rd hit single cell sorted clones and 1st and 2nd hit cell pools. Phi-X marker is positioned in the flanking lanes. Arrow indicates the major uncleaved amplicon detected. **Panel c:** Sanger results from 96 single sorted clones after 3rd hit. All Sanger detected indels from 96 clones are summarized. Note the comparable profile for the indels detected in the pool of cells shown in panel A marked by open arrow heads. **Panel d**: Representative IDAA results from 3rd hit single cell clones displayed in panel b. Notable only one wt allel was detected.

Supplementary Figure 3 │ Indel detection by enzyme mismatch cleavage (EMC) and IDAA.

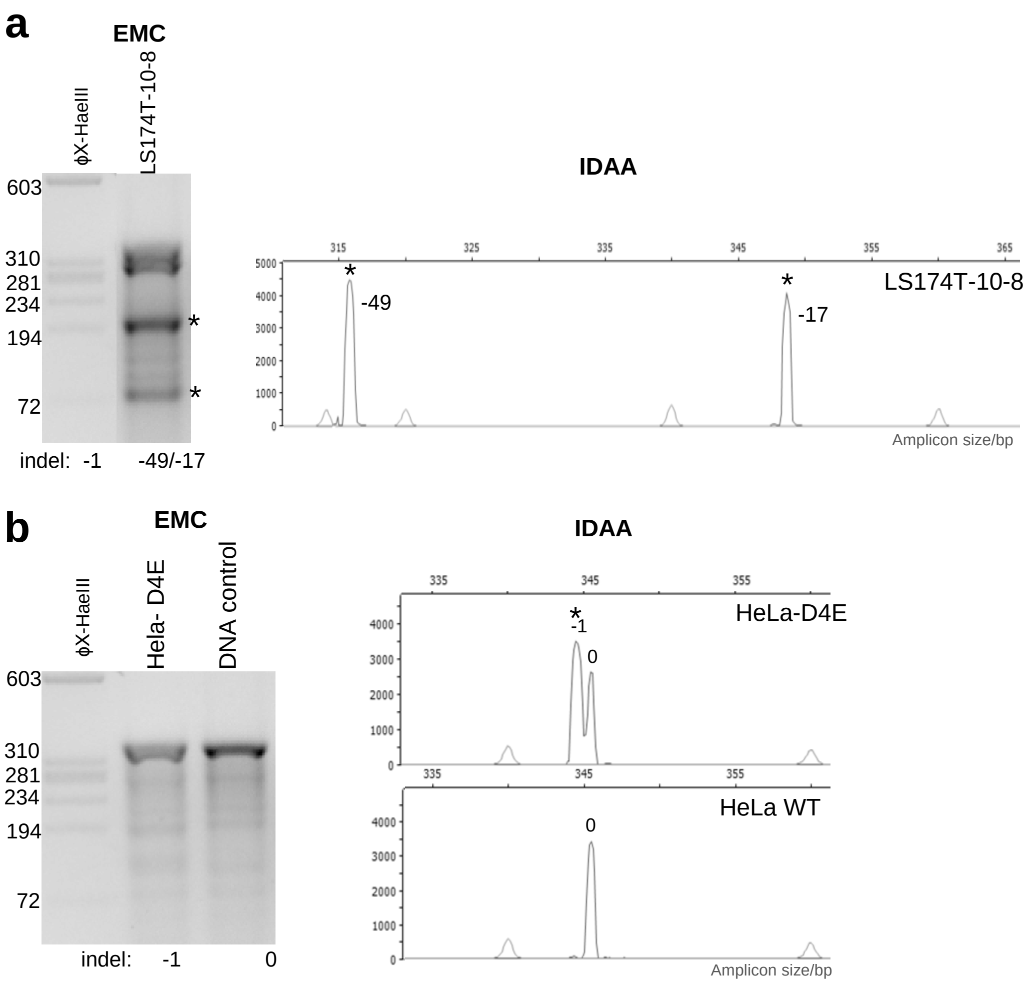

Comparative EMC and IDAA analysis of ZFN targeted clones with large indels or single base indel. EMC assays are commonly based on T4 endonuclease VII (T4E7), endonuclease V (EndoV), T7 endonuclease I (T7EI), CELI or Surveyor nuclease. Panel **a:** EMC (T7EI) assay of amlicons derived from a single LS174T clone (#10-8) targeted with Dual-*GALNT6*-ZFN. Cleaved products are indicated with asterix. Comparative IDAA of the same clone shown to the right. **Panel b:** EMC (T7EI) assay of mixed amplicons derived from a single *COSMC* allele HeLa clone (DE4) (22) and wt HeLa cells targeted with *COSMC*-ZFNs. Comparative IDAA of the same clone demonstrating a monoallelic -1bp deletion (indicated with and asterix) shown to the left, relative to the intact HeLa WT peak(0). Unmarked minor light grey peaks represent the GSLIZ600 standard.

Supplementary Figure 4 │ IDAA of the top off-target candidate site in 10 single cell clones.

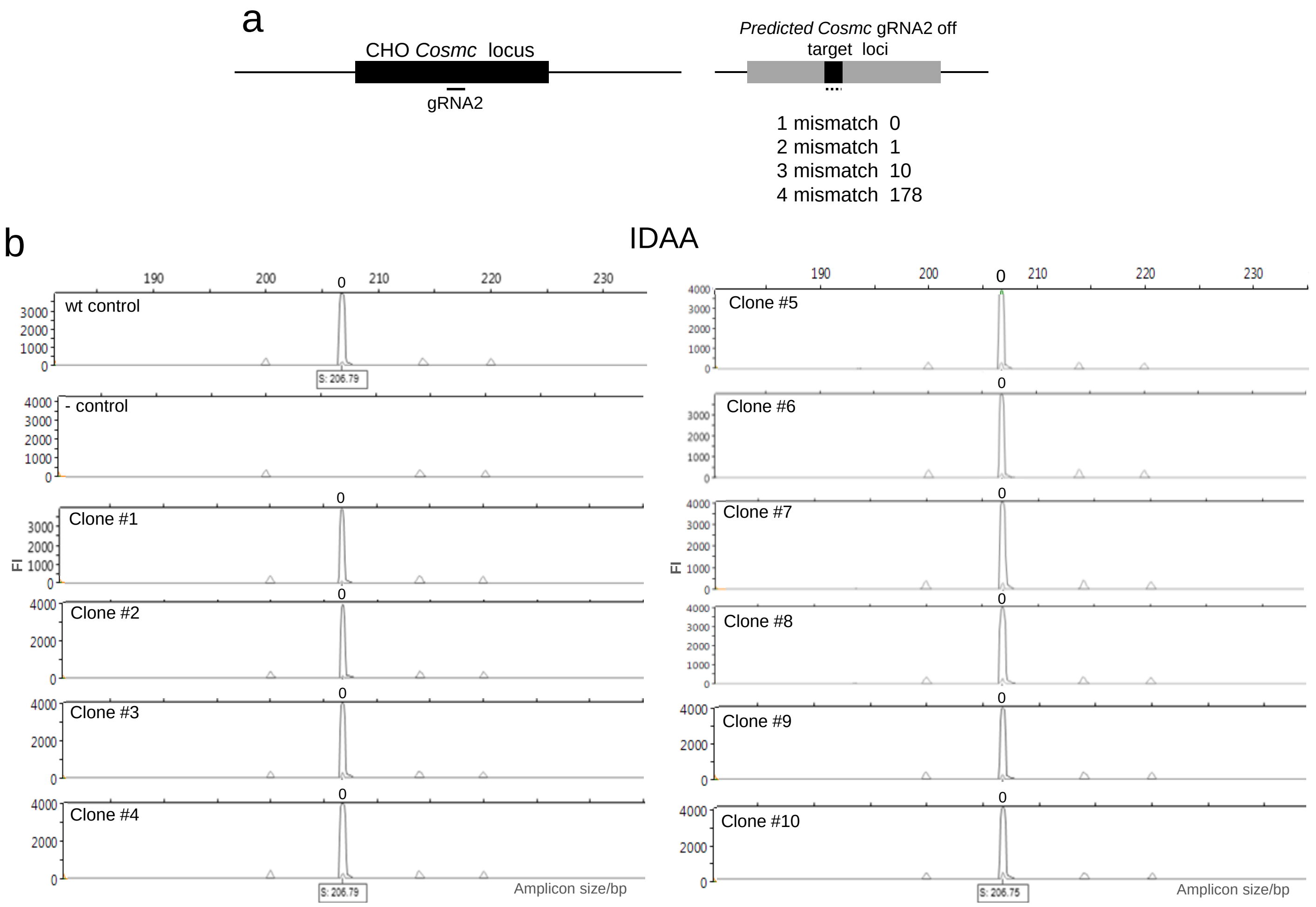

Panel a: Schematic depiction of the gRNA2 target region in the *Cosmc* gene locus and candidate off-target loci with 1-4 mismatches. **Panel b:** IDAA of the top off-target candidate region with 2 mismatches in 10 single CHO cell clones. The *Cosmc* Cas9/gRNA2 targeted single cell sorted clones were from the experiment presented in Figure 2. Position of the intact amplicon indicated by 0 above peak (0bp indel). No off-target events were detected and this was confirmed by Sanger sequencing. LIZ600 marker positions are shown as unmarked peaks within diagrams.

Supplementary Figure 5│ IDAA of top 20 off-target candidate sites in CHO clones.

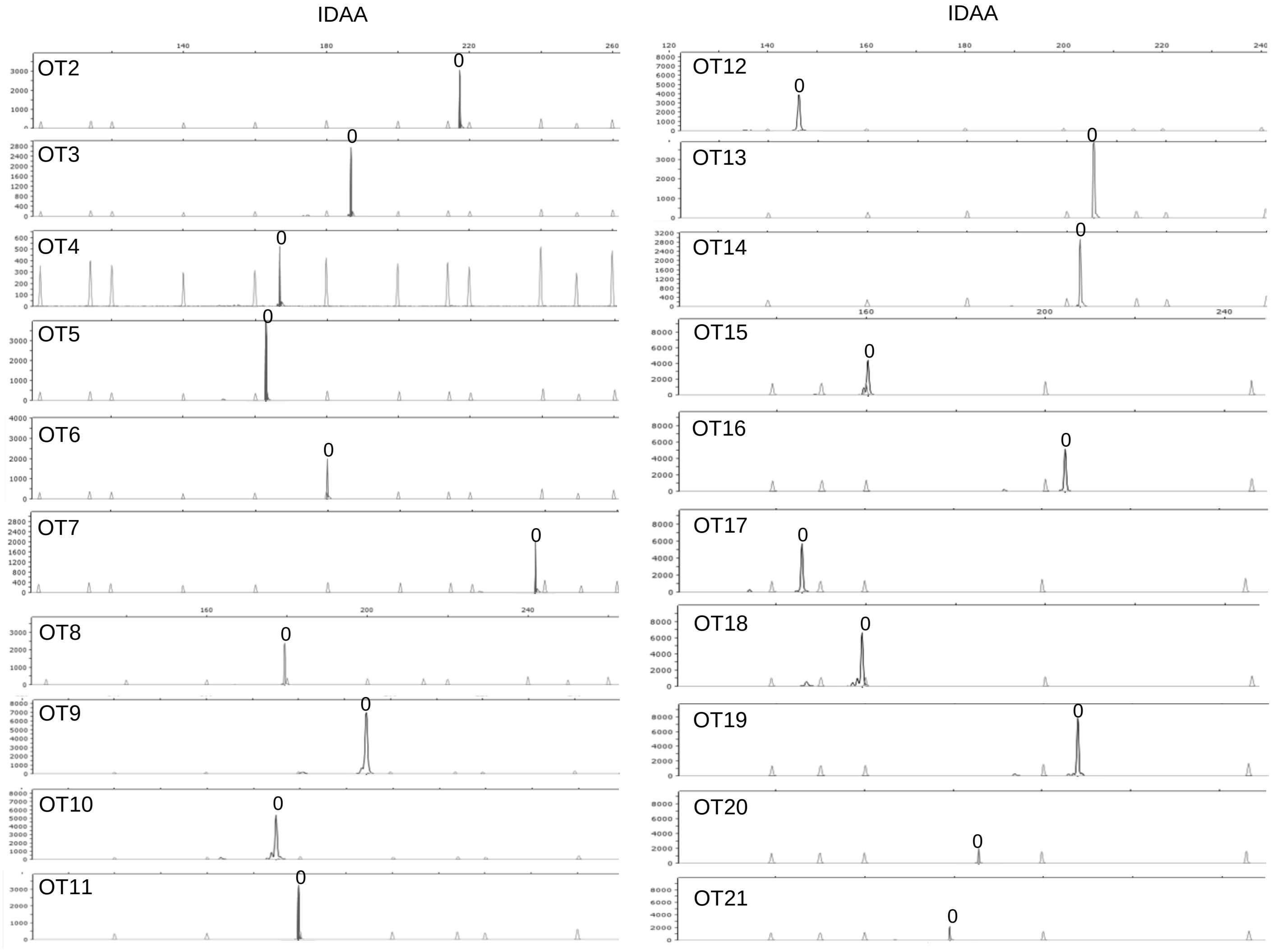

IDAA of the top 21 off-target (OT) loci are shown for a single CHO clone (#7) targeted with *Cosmc* Cas9-gRNA2 from the experiment described for Figure 2. IDAA of all 21 off-targets was performed on additional 9 CHO clones, and no off-target events were identified. IDAA OT1 results are shown in Supplementary Figure 4. Position of the only detected intact amplicon (representing the unmodified off target) is indicated by 0 above peak (0bp indel). Results were verified by Sanger sequencing of all amplicons analyzed. LIZ600 marker positions are shown as unmarked peaks within diagrams. Note, that the relative differences in amplicon product yields for the different targets shown, give rise to considerable variation in the intensities of the LIZ600 marker shown as unmarked peaks in the respective chromatograms.

Supplementary Figure 6│ Cell pool NGS analysis, D2 post transfection.

a

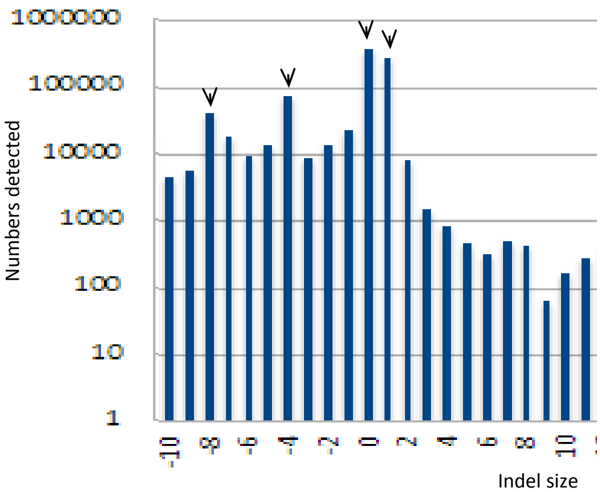

b

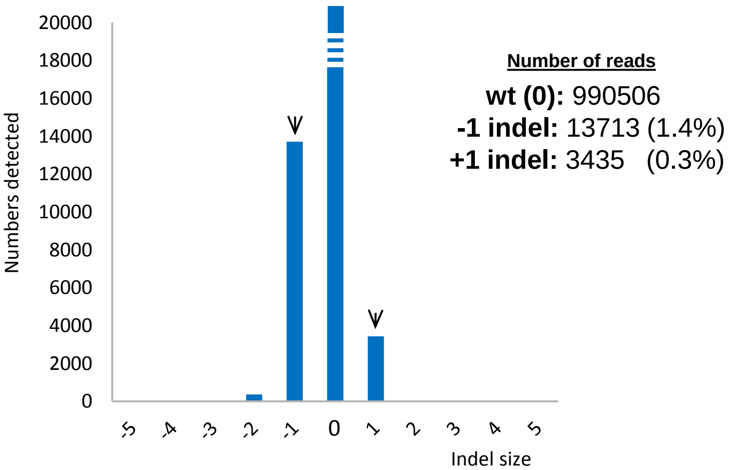

Panel a: CHO cell pool, day 2 post CRISPR/Cas9 *Cosmc* gRNA2 transfection. Number of indels detected with indel sizes ranging from -10bp to +10bp are shown. Note the profile for the -4bp, 0, +1 bp matches the IDAA profiles shown in Figure 2a. Y-axis is logarithmic**. Panel b:** Human HepG2 cell pool, day 2 post Dual-*GALNT6*-ZFN transfection. Number of indels detected with indel sizes ranging from -5bp to +5bp are shown. Note the profile and indel frequencies for the -1bp, 0, +1 bp matches the IDAA profiles shown in Figure 4c.
